# Determining Effective Methadone Doses for Individual Opioid-Dependent Patients

**DOI:** 10.1371/journal.pmed.0030080

**Published:** 2006-02-07

**Authors:** Jodie A Trafton, Jared Minkel, Keith Humphreys

**Affiliations:** **1** Center for Health Care Evaluation, VA Palo Alto Health Care System and Stanford University School of Medicine, Menlo Park, California, United States of America; **2** Department of Psychology, University of Pennsylvania, Philadelphia, Pennsylvania, United States of America; University of QueenslandAustralia

## Abstract

**Background:**

Randomized clinical trials of methadone maintenance have found that on average high daily doses are more effective for reducing heroin use, and clinical practice guidelines recommend 60 mg/d as a minimum dosage. Nevertheless, many clinicians report that some patients can be stably maintained on lower methadone dosages to optimal effect, and clinic dosing practices vary substantially. Studies of individual responses to methadone treatment may be more easily translated into clinical practice.

**Methods and Findings:**

A volunteer sample of 222 opioid-dependent US veterans initiating methadone treatment was prospectively observed over the year after treatment entry. In the 168 who achieved at least 1 mo of heroin abstinence, methadone dosages on which patients maintained heroin-free urine samples ranged from 1.5 mg to 191.2 mg (median = 69 mg). Among patients who achieved heroin abstinence, higher methadone dosages were predicted by having a diagnosis of posttraumatic stress disorder or depression, having a greater number of previous opioid detoxifications, living in a region with lower average heroin purity, attending a clinic where counselors discourage dosage reductions, and staying in treatment longer. These factors predicted 42% of the variance in dosage associated with heroin abstinence.

**Conclusions:**

Effective and ineffective methadone dosages overlap substantially. Dosing guidelines should focus more heavily on appropriate processes of dosage determination rather than solely specifying recommended dosages. To optimize therapy, methadone dosages must be titrated until heroin abstinence is achieved.

## Abstract 2

The dose of methadone associated with heroin abstinence varies greatly between patients. Effective and ineffective dose ranges overlap substantially, and a patient-specific approach to dose determination seems appropriate.

## Introduction

Methadone maintenance is one of the most highly researched and evidence-based treatments for illicit drug dependence. Randomized trials demonstrate that prescribing higher dosages of methadone leads to greater reductions in heroin use in opioid-dependent patients [ 1– 4]. These findings support recommendations that methadone maintenance patients receive daily dosages of over 60 mg per day [ 5]. Yet, some clinicians debate these recommendations and insist that many patients have positive outcomes with lower dosages [ 6]. Consequently, some clinicians reject the research-based recommendations as inconsistent with their clinical experience, and methadone dosing practices vary widely [ 7, 8].

Research and clinical evidence may both be informative. Randomized trials investigate mean population response to a given dosage of methadone, whereas clinicians treat individuals. Examining mean response may de-emphasize the range of individual responses in the population. Additionally, when these studies find that higher dosages of methadone produce greater reductions in heroin use, they tell us that there is a subpopulation of patients who require the higher dosage; they do not provide information about the lowest effective dosage for individuals. Although randomized trials are useful for identifying the active range of the dose response curve for drug effects, they do not specify the optimal dosage for any given individual.

Studies of individual responses to methadone dosages have high potential for translation into clinical practice. Understanding the typical range of dosages over which patients respond may guide physicians in their prescribing practices. Identifying factors related to response at higher or lower dosages may help clinicians predict appropriate dosages for individuals.

Needing a higher dosage of methadone to eliminate heroin use indicates a tolerance to the effects of opioids. Basic science studies suggest a number of factors that may influence individual differences in opioid tolerance, and these were used to guide selection of measured variables in this study. For example, more days of exposure to opioids and higher opioid dosages have been associated with increased opioid tolerance [ 9– 11]. More counterintuitively, withdrawal from opioids may produce sensitization of opioidergic neuronal pathways that may later manifest as opioid tolerance if opioid use is reinstated [ 12, 13]. Genetic background also may modify the development of tolerance [ 14]. Finally, disease states, especially diseases that affect opioidergic systems, may encourage opioid tolerance or sensitivity. For example, pain disorders produced by nerve injury are known to increase tolerance to the analgesic effects of opioids [ 15, 16], and opioid-dependent patients with Axis I psychiatric diagnoses generally need higher methadone dosages [ 17]. There is indication of opioidergic changes in depression and posttraumatic stress disorder (PTSD) as well: decreased opioidergic transmission has been associated with sadness [ 18], case studies have shown that opioids reduce depression symptoms in some patients [ 19, 20], and PTSD is associated with enhanced release of endogenous opioids [ 21– 23].

Treatment delivery factors might also influence the need for higher dosages of methadone. For example, counseling services might reduce drug craving and thus decrease the dosage of methadone needed to produce abstinence. Treatment delivery factors might also limit the population of patients who receive a high enough dosage to abstain. At a clinic that discourages the use of higher methadone dosages, only patients who have lower methadone needs may achieve abstinence.

Factors associated with opioid tolerance have not been well studied in human patients. Determining which individual and treatment delivery variables are associated with increased methadone need in real-life clinical samples will inform the therapeutic use of opioids.

Here we examine methadone dosing practices at eight methadone maintenance clinics run in cities across the United States by the Veterans Health Administration (VA). First, we examined the methadone dosages at which patients stopped heroin use and then compared these effective dosing practices to overall dosing practices at VA clinics. Second, we investigated variables associated with individual differences in the methadone dosage at which each patient achieved heroin abstinence to identify factors that contribute to medication tolerance in opioid-dependent patients.

## Methods

### Recruitment

Patients were recruited through opioid substitution treatment programs located in eight cities throughout the United States. This study is part of the Multi-Site Opioid Substitution Treatment (MOST) study [ 24]. The MOST study was designed to examine differences in patient outcome based upon naturalistic differences in treatment provision. Thus, eight clinic sites were chosen based on their typical treatment practices. Four sites (high guideline-adherent sites) regularly dosed patients above the recommended 60 mg/d minimum methadone dosage; the other four (low guideline-adherent sites) dosed a significant percentage of patients below the recommended 60 mg/d level.

All MOST study procedures were approved by the Institutional Review Board (IRB) of Stanford University and each participating VA medical center (see Protocol S1). All patients provided written informed consent (see Protocol S2). Patients completed a structured interview by telephone at treatment entry and 6 and 12 mo after the start of treatment. Additionally, patients' medical records were accessed for the duration of the study period.

### Sample

Patients in the MOST study were 254 veterans initiating methadone/levo-α-acetyl-methadol (LAAM) treatment between November 2000 and October 2001. Although 267 patients agreed to participate, some dropped out of treatment before receiving a single methadone dose or completing their baseline interview; these participants' data are excluded from this analysis. During the 30 d prior to entering treatment, 86.7% used heroin, 16.1% used illicitly obtained opioid medications, 49.4% used cocaine, 45.9% used alcohol, 21.6% used cannabis, and 10.2% used illicitly obtained sedatives. Historically, 66.8% reported regular use of only heroin, 30.9% reported regular use of heroin and illicit prescription opioids, and 2.3% reported only regular use of illicit prescription opioids. Patients were starting opioid substitution treatment at the time of study entry and thus received low initial dosages that were titrated up through the early phase of treatment. After achieving their first month of abstinence, 55% of patients did not change dosage, 16% decreased dosage, and 29% increased dosage by more than 5 mg during the period in which they maintained abstinence.

Several of the participating clinics also offered opioid substitution with LAAM; typically, LAAM was used in combination with methadone to eliminate the need for patients to come to clinic over weekends. Over the course of treatment, 87.4% of patients received only methadone, 2.4% of patients received only LAAM, and 10.2% of patients received both methadone and LAAM during the course of treatment. Data were originally analyzed in two ways: (1) excluding all patients who received any LAAM, and (2) including the whole sample by converting LAAM dosages to methadone equivalents. The conclusions of both analyses were identical. Because LAAM is no longer manufactured, we present the results from the subpopulation of patients who received only methadone (analysis of the entire population is available upon request from the corresponding author). Thus, the final sample consisted of 222 enrolled patients, 168 of whom achieved at least 1 mo of heroin abstinence and were included in the analysis of factors predicting the dosage of methadone that was effective.

### Measures

Patients completed the Addiction Severity Index (ASI [ 25]) and the SF-36V [ 26] by telephone interview with trained staff members. Data on participants' visits to the VA health care system from October 2000 through September 2003 were downloaded from the national VA databases. Patients' medical and psychiatric diagnoses were extracted from these databases, based upon ICD-9 codes assigned during each medical visit. Patients' dose of methadone for each day in treatment and urinalysis test results were obtained. Case managers of patients in the study were surveyed with an 81.6% response rate regarding their opinions on methadone dosing practices. Questions about case managers' likelihood of suggesting a dosage decrease or detoxification for an abstinent patient were averaged at the clinic level to provide a measure of tendency to discourage use of high dosages at each clinic.

Urinalysis results were obtained from patient medical records. Because this was an observational study, urine screening was conducted at the discretion of the participating clinics, and timing and frequency of testing varied across patients. However, all clinics required that a minimum one urine screen per month be conducted on each patient. The mean number of urine screens per month was 2.86, the median was 4, and the range was 1–13.

Effective dosages were calculated for each patient who had at least 1 mo of abstinence from heroin based upon urinalysis results (i.e., 1 mo where urine tests were performed, and all urine tests were negative for illicit opioids). Mean dosage of methadone was calculated for each month of heroin abstinence for each patient. For patients who had more than 1 mo of abstinence, effective dosage was averaged over abstinent months. Because of concern that differences in treatment trajectory (e.g., attempts to reduce dosage) after patients achieved abstinence might alter study findings, all analyses were also run using average dosage over the first month of abstinence as a measure of effective dosage. The dosage during the first month of abstinence was highly correlated with the mean dosage over all abstinent months (Pearson's correlation coefficient = 0.868, *p* < 0.001), and using first month dosages did not alter the findings of the study. Thus, only results for mean dosage over all abstinent months are presented.

The amount of heroin used per day was estimated from self-reported pretreatment monthly cost of drug use, using average heroin price and purity data from the year of the study for each locality [ 27] and correcting for route of administration [ 28]. In this sample, heroin was administered intravenously by 68%, nasally by 30%, via smoking by 1%, and via nonintravenous injection by 1%.

### Data Analysis

To examine effective versus actual dosing practices, cumulative dosage histograms were created for (1) the subpopulation that had at least 1 mo abstinence from heroin, (2) the subpopulation that never had a month of abstinence from heroin, (3) the subpopulation that attended a clinic with high guideline adherence, (4) the subpopulation that attended a clinic with lower guideline adherence.

In the subpopulation that achieved at least 1 mo of abstinence from heroin ( *n* = 168), Pearson's correlation coefficients (for continuous variables) or point biserial correlation coefficients (for dichotomous variables) were assessed to identify associations between the dosage received while abstinent from heroin and potential tolerance-related factors. A multivariate linear regression model assessed factors that were associated with the dosage at which a patient abstained, controlling for multiple predictors simultaneously.

## Results

### Description of Effective and Actual Dosing Practices

**Figure 1:**
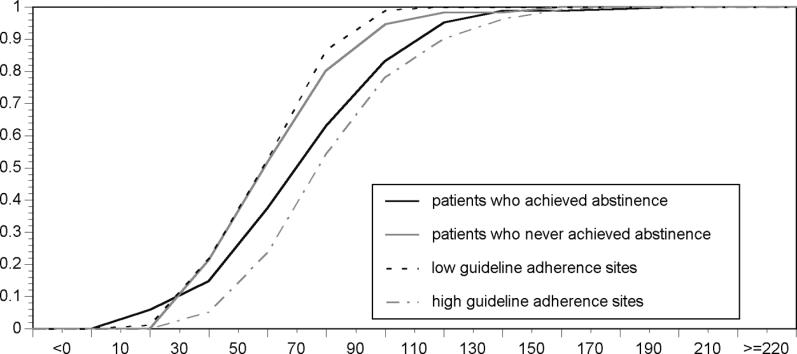
Cumulative Dose Histogram Depicting the Proportion of Patients at or below a Given Methadone Dose (in mg) Black line: methadone doses received while patients maintained heroin abstinence ( *n* = 168). Gray line: average methadone doses received by patients who never achieved heroin abstinence ( *n* = 54). Black dashed line: average methadone doses received by patients at low guideline-adherence clinics ( *n* = 88). Gray dashed line: average methadone doses received by patients at high guideline-adherent clinics ( *n* = 134).

One hundred sixty-eight patients had at least 1 mo of abstinence from heroin. Effective methadone dosages ranged from 1.5 to 191.2 mg, the median effective dosage was 69 mg ( Figure 1). Notably, 38% of patients who were heroin abstinent for a month did so on less than 60 mg methadone (i.e., below the minimum daily dosage recommended in clinical practice guidelines). In contrast, 16% of patients required over 100 mg. The 25% of patients who did not achieve abstinence received a mean dosage of 61 mg methadone (range: 20–150 mg; median: 60 mg). Of patients who did not achieve abstinence, 45.6% received over 60 mg methadone.

Mean daily dosages of methadone for patients in treatment ranged from 30 to 167 mg (median: 76.3 mg) at high guideline-adherent sites ( *n* = 134) and from 20 to 100 mg (median: 59.7 mg) at low guideline-adherent sites ( *n* = 88).

Mean daily dosages received by patients who did not abstain from heroin use and mean daily dosages at low guideline-adherent sites were left-shifted on the cumulative dosage histogram as compared to effective dosages, suggesting that these patients were being dosed lower than necessary. This difference was particularly pronounced in the higher dosage ranges. In contrast, dosages at high guideline-adherent sites were right-shifted as compared to effective dosing, suggesting that patients were receiving just slightly higher dosages than those given to patients who achieved abstinence. This variation from effective dosing practices is consistent with heroin use outcomes observed at high versus low guideline-adherent sites. Patients who attended high guideline-adherent clinics had significantly lower rates of heroin use at follow-up [ 24].

### Correlations between Patient Factors and Methadone Tolerance

To determine factors associated with tolerance to the effects of methadone on heroin use, we examined whether quantity and frequency of drug use, experience of opiate withdrawal, family history of substance abuse, disease states, or treatment delivery factors were correlated with the dosage of methadone at which patients achieved heroin abstinence ( Table 1).

**Table 1:**
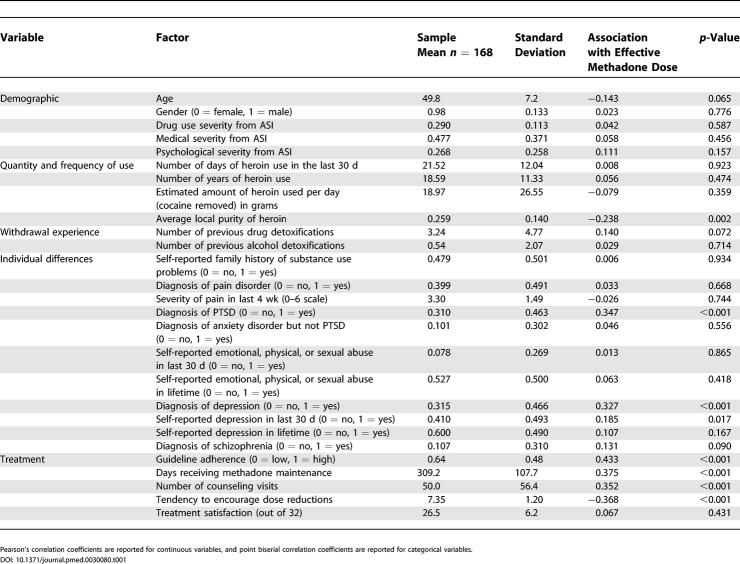
Associations between Potential Opioid Tolerance-Related Factors and the Methadone Dose at Which Patients Achieved Heroin Abstinence

### Use-Dependent Factors

#### Quantity and frequency of use

Recent frequency of heroin use, the number of years that heroin had been used, and estimates of the amount of heroin used per day were not associated with effective methadone dosing levels. Higher local purity of heroin was associated with lower effective methadone dosage; this association was the opposite of common predictions.

#### Withdrawal

Brief periods of withdrawal from opioids increase opioid tolerance in basic science studies [ 12, 13]. We expect that opioid detoxification treatments constitute a significant percentage of the major withdrawal episodes that patients have experienced. We found that the number of previous drug detoxification treatments experienced tended to correlate with the dosage of methadone needed to abstain from heroin. As expected, the number of previous ethanol detoxification treatments was not correlated with the effective methadone dosage.

### Individual Differences

#### Family history

Although we do not have any measure of opioid tolerance in family members, this study did collect patient-reported data on family history of drug and alcohol problems. Having a parent with a drug or alcohol problem was not associated with the effective methadone dosage.

### Disease States

#### Chronic pain

Chronic pain states, such as neuropathy, have been associated with analgesic tolerance to opioid medications [ 15, 16]. To determine if chronic pain conditions increase tolerance to methadone's ability to prevent heroin use, we examined the association between reported pain levels and diagnoses of chronic pain conditions and effective methadone dosage. The severity of pain experienced in the past 4 wk or having a diagnosis of a chronic pain condition was not correlated with the effective dosage of methadone.

#### PTSD

Having a recent diagnosis of PTSD was strongly correlated to the effective methadone dosage. This association did not hold for other anxiety disorders. Similarly, a recent or lifetime history of emotional, physical, or sexual abuse was not related to methadone tolerance, suggesting that methadone tolerance was related to PTSD, not trauma experience.

#### Depression

We examined whether depressive symptomatology or diagnosis was related to methadone dosing needs. Self-reported depression in the past 30 d and having a recent diagnosis of clinical depression correlated positively with effective dosage. However, self-reported depression experienced throughout the lifetime was not correlated with effective methadone dosage.

#### Schizophrenia

As a control, we examined whether mental health disorders for which specific opioid contributions have not been implicated were related to effective methadone dosage. Schizophrenia was not significantly correlated with effective dosage of methadone.

#### Treatment delivery factors

Patients attending clinics designated as high guideline-adherent by the MOST study maintained abstinence on higher dosages of methadone (high: 77.0 ± 30.5 mg; low: 52.4 ± 24.7 mg; *p* < 0.001). To identify specific treatment factors contributing to this correlation, we examined the relationship of effective dosage with length of treatment, number of counseling visits, satisfaction with treatment, and the tendency of a clinic's counselors to encourage abstinent patients to reduce or eliminate their dosage of methadone. Patients who stayed in treatment longer, received more counseling visits, and attended clinics where counselors did not encourage dose reduction received higher methadone dosages during periods of abstinence ( Table 1).

#### Predictors of methadone tolerance

To determine the predictive value of the above factors, patient variables significantly associated with methadone dosage were included in a multivariate linear regression model with effective methadone dosage as the dependent variable. Because the study examined patient outcomes from sites specifically chosen for their differences in treatment practices, a variable encoding whether the patient attended a high or low guideline-adherent treatment clinic was included in the model. Whether the clinic generally followed treatment guidelines, the number of previous drug detoxification treatments the patient had completed, whether the patient had a diagnosis of PTSD or depression, and the local purity of heroin predicted the dosage at which patients abstained from heroin use ( *R* ^2^ = 0.335, *p* < 0.001). To assess which aspects of guideline adherence were related to effective methadone dosing, we replaced the variable encoding guideline-adherence rate with treatment-level variables found to correlate with effective dosage. The modified model better predicted the effective methadone dosage ( *R* ^2^ = 0.423, *p* < 0.001). The patients who remained in methadone maintenance treatment for a longer period of time and attended clinics where counselors reported not favoring dosage reductions in abstinent patients received higher effective methadone dosages. The number of counseling visits attended was correlated with the length of time patients stayed in treatment and did not additionally predict effective methadone dosage ( *p* > 0.05). Having a diagnosis of PTSD increased effective methadone dosage by 12.1 mg (95% confidence interval: 3.4–20.8 mg). A diagnosis of depression also increased effective methadone dosage by 14.1 mg (95% confidence interval: 5.6–22.6 mg). Each previous drug detoxification episode increased the dosage of methadone at which patients abstained from heroin by 0.9 mg (95% confidence interval: 0.1–1.7 mg). For every 10% increase in local heroin purity, the methadone dosage needed to maintain abstinence decreased by 4.0 mg (95% confidence interval: −6.8 to −1.2 mg). Patients who were abstinent from heroin received 0.1 mg more (95% confidence interval: 0.06–0.14 mg) for each day they remained in treatment. For each point change on the counselor's tendency to encourage dosage reduction scale, patients received 7.4 mg (95% confidence interval: −10.5 to −4.2 mg) less methadone while abstinent from heroin. In total, these six variables accounted for 42.3% of the variance in the dosage of methadone at which patients abstained.

Adding common predictive variables to the model did not alter the overall findings of the above model. Age, race, baseline medical severity, or baseline substance use severity did not significantly predict effective methadone dosage ( *p* >.10) and did not eliminate the significance of the predictive value of the other six variables.

## Discussion

### Dosing Curves

Individual patients' opioid substitution medication needs vary greatly, a fact that is often overlooked in treatment research. The dosages typically tested in randomized trials of methadone maintenance to date are below the dosage needed to achieve abstinence in patients at the high end of the dose response curve [ 1, 3, 4, 6]. Thus, higher dosage conditions in trials almost always produce better outcomes on average than do lower dosage conditions.

The cumulative dose-response curve lends credence to clinicians' insistence that methadone dosing guidelines do not apply to all patients. Fully 40% of patients in this study maintained heroin abstinence on less than the recommended 60 mg methadone per day. Nevertheless, the importance of adequate dosing is obvious, as the slight leftward shift of the methadone dosage histogram in low guideline-adherent sites was associated with significantly greater heroin use among patients. These data suggest that clinicians should be allowed significant flexibility in methadone dosing as long as outcomes are positive because extremely low dosages (2 mg/d) may be effective in some patients, while others require extremely high dosages (over 160 mg/d) to abstain from heroin. Minimum dosage requirements are not necessary; however, clinicians must recognize that a subset of patients will be underdosed at dosages as high as 140 mg/d. Dosage titration utilizing drug screening to measure dosage effectiveness should facilitate the determination of appropriate dosages for individual patients.

### Individual Factors

Our analysis of factors related to opioid tolerance can inform clinicians' predictions of individual patients' methadone needs. First, these results suggest several possibly counterintuitive practices relating to patients' opioid use history. Although it may seem logical to expect patients who used lesser amounts or less pure heroin to need lower dosages of methadone, this expectation is not supported by our data. The amount of heroin used per day did not predict effective methadone dosage, and living in an area with lower average local heroin purity predicted need for higher methadone dosages. It cannot be assumed that patients who became dependent using lower dosages of heroin will need lower dosages of methadone to achieve abstinence.

Ibuki et al. [ 12, 13] hypothesized that excessive neuronal firing during opioid withdrawal facilitates signaling through opioidergic pathways; this sensitization may later manifest as tolerance to the effects of opioids. This theory predicts the counterintuitive result identified here that repeated opioid detoxifications increase tolerance to opioids. This may also explain the finding that low local heroin purity is associated with greater methadone needs. Assuming those using less pure heroin achieve lower blood levels of opioid with each use, users in areas with low local heroin purity would experience withdrawal more quickly after each use and thus more frequently over time.

Willenbring and colleagues [ 29] observed that methadone clinics with low patient turnover and a large number of patients who had been maintained stably in treatment for years had successful treatment outcomes on lower average methadone dosages. Keeping patients on stable opioid dosages and limiting periods of withdrawal (e.g., repeated attempts to cease or reduce methadone use) may reduce development of opioid tolerance and allow for successful long-term treatment of opioid dependence with low dosages of methadone. These results suggest that maintaining a patient on a dosage at which he or she experiences withdrawal symptomatology late in the dosage cycle, or repeatedly attempting to withdraw a patient from methadone, will not limit the need for methadone and may in fact increase the dosage of methadone needed to achieve abstinence over the long term.

Although it has been reported that some opioid substitution treatment clinics provide more methadone to their patients with chronic pain [ 30], this did not appear to be necessary to improve abstinence rates in our sample. Even though chronic pain may be treated with opioid therapy, patients with higher pain levels did not require more methadone to abstain from heroin use.

In contrast, having a diagnosis of depression or PTSD was a strong predictor of need for higher dosages of methadone. Our results suggest that patients with these conditions should be expected to require higher medication dosages, and thus, it may make sense to more aggressively titrate dosages early in treatment in these patients.

### Treatment Delivery Factors

Although treatment factors are less likely to explain individual differences in methadone dosage needs, they likely influence who receives an adequate dosage to achieve abstinence. Treatment factors that result in reduced likelihood of receiving an adequate dosage will bias the population that achieved abstinence toward those who require lower methadone dosages. Thus, dropping out of treatment at an earlier time and attending a clinic that encourages dosage reductions is associated with lower effective methadone doses. We believe that only those patients with low tolerance to methadone achieve abstinence early in treatment or when clinicians encourage reductions in dosages. Encouraging rapid dose titration early in treatment and discouraging attempts at dosage reduction or cessation should improve the percentage of patients who achieve abstinence.

### Limitations of This Study

The results reported in this study are predictive associations, but causation cannot be assumed. Also, the clinics participating in this study treated few women and younger patients. This may limit generalizability of the results. Although including patients who received LAAM during treatment did not change the study results, our analysis is not sufficient to conclude that the results of this study can be generalized to opioid medications other than methadone (e.g., LAAM or buprenorphine). Finally, because the study was observational, we cannot know that the dosage a patient received while abstinent was the minimal dosage required for abstinence. Some patients might have maintained abstinence on lower dosages.

### Future Directions

We suggest that future treatment research investigate processes of dosage determination rather than specific drug dosages (e.g., compare two treatment strategies; see [ 31] for an example). When there is large variation in individual dose-response relationships, overall dosage-level recommendations may not provide clinicians with sufficient information to guide treatment practice. We suggest that research that identifies the most effective process for determination of medication dosage may be more effectively translated into clinical practice.

### Summary

The range of effective methadone doses for treatment of opioid dependence is broad, and treating clinicians should titrate doses to full effect in each individual patient. Dosing guidelines should include advice on appropriate processes of dosage determination. Patients with PTSD, depression, numerous prior opioid detoxification treatments or withdrawal episodes, and those who use low-purity heroin are likely to require higher dosages of methadone to achieve abstinence.

## Supporting Information

Protocol S1 IRB Approval Letter from the Stanford University IRB for Oversight of the MOST Study at the VA Palo Alto (182 KB PDF).

Protocol S2 Sample Consent Form This is a sample of a consent form from one of our eight participating research sites. Consent forms from the other seven participating sites were similar, with minor modifications of formatting and language to comply with local IRB preferences.
(76 KB PDF).

**Patient Summary**

**Background**

Methadone is a legal and rigorously tested synthetic drug that acts on the same brain targets as heroin. Because methadone lasts a lot longer in the body than heroin, patients on methadone do not experience the extreme highs and lows that are felt by people who use heroin (the highs and lows result from the waxing and waning of heroin levels in the blood). Methadone has been used for more than 30 years to help patients overcome heroin addiction. If patients take methadone as prescribed, they are unlikely to get withdrawal symptoms when they stop taking heroin, or the withdrawal symptoms are much less severe. Additionally, methadone blocks the high produced by heroin so that taking heroin is no longer rewarding. Patients on methadone remain physically dependent on the drug, but most of them no longer have the uncontrolled, compulsive, and disruptive behavior caused by heroin addiction. Prolonged methadone treatment under medical supervision has been shown to be safe, and it allows patients to lead “normal” lives while taking a daily dose of methadone.

**Why Was This Study Done?**

In many studies over the past decade or so, researchers have tried to determine which methadone dosages work best to help patients overcome heroin addiction. Based on these studies, dosages of at least 60 mg/day are recommended. That said, doctors in methadone clinics often see a wide range of dosages that are effective, and some of them treat patients with dosages that are lower or higher than the recommended amount. In this study, the researchers examined the range of methadone dosages that helped patients achieve heroin abstinence. They also wanted to find factors that influenced whether a particular patient needed a higher or a lower dosage.

**What Did the Researchers Do and Find?**

They studied 222 heroin-addicted volunteers who started methadone treatment and followed them for a year. Of these, only 168 achieved at least one month of heroin abstinence. The range of effective methadone dosages (among the patients who achieved abstinence) was very wide, from 1.5 mg to 191.2 mg. Thirty-eight percent of the patients achieved abstinence on less than 60 mg, and 16% of the abstinent patients received a dosage of over 100 mg. On the other hand, almost half of the patients who did not achieve heroin abstinence received the recommended dosage of 60 mg or more methadone. Overall, patients at clinics that generally adhered to the treatment guidelines (and treated most patients at 60 mg or higher) were more likely to achieve abstinence. Among patients who achieved abstinence, higher methadone doses were correlated with posttraumatic stress disorder, depression, a higher number of previous detoxifications from heroin, and some other factors.

**What Does This Mean?**

This study confirms that effective methadone doses (as defined by heroin abstinence for at least a month) vary very widely. It shows that even high dosages that work for one patient may be too low for another patient and that a substantial fraction of patients achieve abstinence on less than the recommended dosage. The results also suggest that there are some factors that might predict whether a patient is more likely to need a higher dosage, such as a diagnosis of posttraumatic stress disorder or depression. Most surprisingly, the results suggest that attempts to stop using methadone may actually increase the need for methadone over the long term. It seems therefore reasonable that doctors monitor the effects of treatment in each individual patient to find the most effective dose for that individual. However, as patients in clinics that adhere to the guidelines do better on average, the recommended dose should serve as a benchmark that might be adjusted upward or downward.

**Where Can I Find More Information Online?**

The following Web sites contain information on methadone therapy for heroin addiction.

MedlinePlus:

http://www.nlm.nih.gov/medlineplus/druginfo/medmaster/a682134.html

Omni:

http://omni.ac.uk/browse/mesh/D008691.html

American Association for the Treatment of Opioid Dependence:

http://www.aatod.org

Fact sheet on methadone from the US government: http://www.whitehousedrugpolicy.gov/publications/factsht/methadone

UK Web site on addiction:

http://www.recovery.org.uk/druginfo/index.html

## Abbreviations

ASI: Addiction Severity Index
IRB: Institutional Review Board
LAAM: levo-α-acetyl-methadol
MOST: Multi-Site Opioid Substitution Treatment
PTSD: posttraumatic stress disorder
VA: Veterans Health Administration

## References

[pmed-0030080-b001] Kosten TR, Schottenfeld R, Ziedonis D, Falcioni J (1993). Buprenorphine versus methadone maintenance for opioid dependence. J Nerv Ment Dis.

[pmed-0030080-b002] Strain EC, Stitzer ML, Liebson IA, Bigelow GE (1993). Methadone dose and treatment outcome. Drug Alcohol Depend.

[pmed-0030080-b003] Ling W, Wesson DR, Charuvastra C, Klett CJ (1996). A controlled trial comparing buprenorphine and methadone maintenance in opioid dependence. Arch Gen Psychiatry.

[pmed-0030080-b004] Maxwell S, Shinderman M (1999). Optimizing response to methadone maintenance treatment: Use of higher dose methadone. J Psychoactive Drugs 1999.

[pmed-0030080-b005] US Department of Veterans Affairs (2001). Management of substance use disorders in primary and specialty care, v 1.0.

[pmed-0030080-b006] Maremmani I, Pacini M, Lubrano S, Lovrecic M (2003). When “enough” is still not “enough”: Effectiveness of high-dose methadone in the treatment of heroin addiction. Heroin Add & Rel Clin Probl.

[pmed-0030080-b007] D'Aunno T, Pollack HA (2002). Changes in methadone treatment practices: Results from a national panel study, 1988–2000. JAMA.

[pmed-0030080-b008] D'Aunno T, Vaughn TE (1992). Variations in methadone treatment practices: Results from a national study. JAMA.

[pmed-0030080-b009] Stevens CW, Yaksh TL (1989). Potency of infused spinal antinociceptive agents is inversely related to magnitude of tolerance after continuous infusion. J Pharmacol Exp Ther.

[pmed-0030080-b010] Stevens CW, Yaksh TL (1989). Time course characteristics of tolerance development to continuously infused antinociceptive agents in rat spinal cord. J Pharmacol Exp Ther.

[pmed-0030080-b011] Stevens CW, Yaksh TL (1992). Studies of morphine and D-ala2-D-leu5-enkephalin (DADLE) cross-tolerance after continuous intrathecal infusion in the rat. Anesthesiology.

[pmed-0030080-b012] Ibuki T, Dunbar SA, Yaksh TL (1997). Effect of transient naloxone antagonism on tolerance development in rats receiving continuous spinal morphine infusion. Pain.

[pmed-0030080-b013] Ibuki T, Marsala M, Masuyama T, Yaksh TL (2003). Spinal amino acid release and repeated withdrawal in spinal morphine tolerant rats. Br J Pharmacol.

[pmed-0030080-b014] Kest B, Hopkins E, Palmese CA, Adler M, Mogil JS (2002). Genetic variation in morphine analgesic tolerance: A survey of 11 inbred mouse strains. Pharmacol Biochem Behav.

[pmed-0030080-b015] Arner S, Meyerson BA (1988). Lack of analgesic effects of opioids on neuropathic and idiopathic forms of pain. Pain.

[pmed-0030080-b016] Kupers RC, Konings H, Adriaensen H, Gybels JM (1991). Morphine differentially affects the sensory and affective pain ratings in neurogenic and idiopathic forms of pain. Pain.

[pmed-0030080-b017] Maremmani I, Zolesi O, Aglietti M, Marini G, Tagliamonte A (2000). Methadone dose and retention in treatment of heroin addicts with Axis I psychiatric comorbidity. J Addict Dis.

[pmed-0030080-b018] Zubieta JK, Ketter TA, Bueller JA, Xu Y, Kilbourn MR (2003). Regulation of human affective responses by anterior cingulate and limbic mu-opioid neurotransmission. Arch Gen Psychiatry.

[pmed-0030080-b019] Bodkin JA, Zornberg GL, Lukas SE, Cole JO (1995). Buprenorphine treatment of refractory depression. J Clin Psychopharmacol.

[pmed-0030080-b020] Cohen MR, Pickar D (1981). Pharmacological challenges to the endogenous opioid system in affective illness. J Clin Psychopharmacol.

[pmed-0030080-b021] Baker DG, West SA, Orth DN, Hill KK, Nicholson WE (1997). Cerebrospinal fluid and plasma beta-endorphin in combat veterans with post-traumatic stress disorder. Psychoneuroendocrinology.

[pmed-0030080-b022] van der Kolk BA, Greenberg MS, Orr SP, Pitman RK (1989). Endogenous opioids, stress induced analgesia, and posttraumatic stress disorder. Psychopharmacol Bull.

[pmed-0030080-b023] van der Kolk B, Greenberg M, Boyd H, Krystal J (1985). Inescapable shock, neurotransmitters, and addiction to trauma: Toward a psychobiology of post traumatic stress. Biol Psychiatry.

[pmed-0030080-b024] Humphreys K, Trafton J, Barnett P (2003). Final report for HSR&D project SUS 99–026–2: Clinical practices and outcomes in VA methadone programs.

[pmed-0030080-b025] McLellan AT, Arndt IO, Metzger DS, Woody GE, O'Brien CP (1993). The effects of psychosocial services in substance abuse treatment. JAMA.

[pmed-0030080-b026] Kazis LE, Ren XS, Lee A, Skinner K, Rogers W (1999). Health status in VA patients: Results from the Veterans Health Study. Am J Med Qual.

[pmed-0030080-b027] Drug Enforcement Administration (2002). Domestic Monitor Program. DEA report # 03057.

[pmed-0030080-b028] Hendriks VM, van den Brink W, Blanken P, Bosman IJ, van Ree JM (2001). Heroin self-administration by means of “chasing the dragon”: Pharmacodynamics and bioavailability of inhaled heroin. Eur Neuropsychopharmacol.

[pmed-0030080-b029] Willenbring ML, Hagedorn HJ, Postier AC, Kenny M (2004). Variations in evidence-based clinical practices in nine United States Veterans Administration opioid agonist therapy clinics. Drug Alcohol Depend.

[pmed-0030080-b030] Peles E, Schreiber S, Gordon J, Adelson M (2005). Significantly higher methadone dose for methadone maintenance treatment (MMT) patients with chronic pain. Pain.

[pmed-0030080-b031] Havassy B, Hargreaves WA (1979). Self-regulation of dose in methadone maintenance with contingent privileges. Addict Behav.

