# Supplementary material for: Determining Effective Methadone Doses for Individual Opioid-Dependent Patients
Source: PLoS Med. 2006 Feb 7;3(3):e80. doi: 10.1371/journal.pmed.0030080 (PMC1360079; doi:10.1371/journal.pmed.0030080)
Supplement: Supplementary file 1 [file pmed.0030080.sd001.pdf]

STANFORD UNIVERSITY

Stanford, California 94305-5401

Penny Eckert, Ph. D. (650) 723-2480
CHAIR, PANEL ON NON-MEDICAL HUMAN SUBJECTS (650) 725-8013(fax)
c/o Administrative Panels Office

Certification of Human Subjects Approval

Date: September 24, 2004
To: **Humphreys, Keith** , Department Of Psychiatry & Behavrl Sci
Jodie Trafton , Catherine Orece Hammonds , Whitney Waldroup , Stephen Tracy , Jill Sempel , Genivieve Tregor , Luisa Ladd , Brian Nguyen
From: Penny Eckert, Ph. D., Chair, Administrative Panel on Human Subjects in Non Medical Research
Faculty Sponsor:
Protocol Title:
Clinical Practices and Outcomes in VA Methadone Programs

Protocol ID: 81012 **IRB Number:** 349 (Panel 2)

The Panel approved human subjects involvement in your research project on September 24, 2004. If there is a contract involved, it must be signed before subject recruitment and enrollment.

The expiration date of this approval is September 23, 2005. If this project is to continue beyond that date, please submit an updated proposal in advance for the Panel's re-approval. If this proposal is used in conjunction with any other human experimentation or if it is modified in any way, it must be re-approved for these special circumstances. **Note that all adverse events, occurring here and at other institutions, should be reported to both the Human Subjects and Biosafety Panels regardless of whether or not the events are thought to be related to the gene transfer intervention.**

All continuing projects and activities must be reviewed and re-approved at least annually by the Panel. Panel approval of any project is for a maximum period of one year. It is the responsibility of the investigator to submit quarterly reports to the Panel and to resubmit the project to the Panel for continuing review.

Please remember that all data including all signed consent form documents must be retained for a minimum of three years past the completion of this research. Additional requirements may be imposed by your funding agency, your department, or other entities. (Policy on Retention of and Access to Research Data, Research Policy Handbook, www.stanford.edu/dept/DoR/rph/2-10.html).

This institution is in compliance with requirements of protection of human subjects (45 CFR 46 and 21 CFR 56).

Penny Eckert, Ph. D., Chair

Approval Period: 9/24/2004 through 9/23/2005
Review Type: Regular - RENEWAL
Funding:
Assurance Number FWA00000935 (SU); FWA00000929 (VA)
IRB Number: 349 (Panel 2)
