# Supplementary material for: Determining Effective Methadone Doses for Individual Opioid-Dependent Patients
Source: PLoS Med. 2006 Feb 7;3(3):e80. doi: 10.1371/journal.pmed.0030080 (PMC1360079; doi:10.1371/journal.pmed.0030080)
Supplement: Supplementary file 2 [file pmed.0030080.sd002.pdf]

Subject name: _______________________________________

Title of Study: MULTI-SITE OPIOID SUBSTITUTION TREATMENT STUDY

Principal Investigator: Joseph Liberto, MD (410) 605-7368/7357
Co-Investigators: Phil Eichmiller, LCSW-C,MPH, (410) 605-7288/7357
Kathy Breslin, BS,M.E.ds. (410) 605-7000x6082/605-7357
Mark Mann, MA (410) 05-7000x5526/605-7357

---

I. PURPOSE OF THE STUDY

The Veterans Administration (VA) is conducting a study of the effectiveness of its methadone/LAAM (Levo Alpha Acetyl Methadol) treatment programs. The study will compare the treatment that is provided by the VA at 8 of its program sites. You are invited to participate in this study of methadone/LAAM treatment for your opiate (heroin) addiction.

This study examines how well methadone/LAAM treatment programs benefit individuals who are addicted to heroin. The experiences of patients from the 8 VA programs will be compared to determine which program practices work best. Specifically, the study examines the effect of various VA methadone/LAAM treatment approaches on:

- ongoing heroin and other drug/alcohol usage during treatment;
- the general health and sense of well being during treatment;
- areas of social functioning such as employment, housing, family relations, etc., during treatment.

In addition, the study collects information on how satisfied the heroin addicted are with VA methadone/LAAM treatment. It also examines how much patient improvement there is for the amount of time and money that it takes to provide treatment.

The study examines these questions during your first year of treatment. You are invited to take part in this study during your first year of treatment. You are eligible to be in the study because you are addicted to heroin, seeking VA methadone/LAAM treatment and are eligible for VA care.

Methadone and LAAM are replacement narcotic medicines. They relieve the urge and the need to use heroin. LAAM is similar to methadone but has a longer lasting effect. Because of this longer effect, it can be taken every other day. Methadone is taken daily. At varying times in your treatment, you may either be treated with methadone or LAAM. At some point, you may in fact be treated with a combination of the two medications.

Methadone and LAAM are the standard of care for the heroin addicted. Since you have already been accepted for methadone/LAAM treatment, you will receive the medication and counseling regardless of your decision to enter or stay in the study.

## II. PROCEDURES

If you agree to participate in this study, you will be asked to complete three telephone interviews. Each interview will take approximately 90 minutes to complete.

The first telephone interview will be at admission to the program. It will be a combination of the following: a questionnaire that reviews the history of your addiction and its impact on your psychological and social functioning; a drug usage risk assessment; questions about your use of VA and community medical services; a questionnaire assessing your quality of life; and questions about your degree of satisfaction with the treatment you have received.

The second and third telephone interviews are essentially the same as the first but will take less time to complete. This is because they have less questions. The second interview will be 6 months after you started treatment. The third interview will be one year after you started treatment. These two interviews will need to be completed even if you are no longer involved in VA methadone/LAAM treatment.

The Menlo Park VA Center for Health Care Evaluation will collect results of drug testing and the utilization of VA medical services from the VA computer database. The methadone program will also provide your methadone/LAAM medication dosing records to the study center on an ongoing basis. This gives the study staff the ability to determine which dosing practices (for example higher versus lower dosing) benefit the addicted most.

You will be asked to provide the names and telephone numbers of people that will know how to reach you during the upcoming year. They would be called to try to locate you for a follow up telephone interview if you were unable to be reached through the VA clinic or the telephone number(s) you provided. Providing these names and numbers does not change your right to confidentiality. Neither the nature of your drug problem nor the treatment you are receiving would be disclosed during these calls.

## III. RISKS/DISCOMFORT

There are no obvious risks to participating in this study. While many of the questions about your drug usage and general health may be very personal in nature, they are the very questions/issues that are addressed in standard methadone/LAAM program treatment. If you become upset, there are program counselors and psychiatrists available to help.

## IV. BENEFITS

You will not directly benefit from your participation in this study. The study will help the VA to better understand the degree to which heroin addicted individuals benefit from methadone/LAAM treatment. It should also help the VA to better understand which treatment procedures work best. If the findings are favorable, it may, in the future, lead to a more successful treatment and increased methadone/LAAM availability.

## IV. ALTERNATIVES TO PARTICIPATION

Since this is not a treatment study, your option is not to participate.

V. COST/COMPENSATION

You will be provided $25 compensation for each of the three study telephone interviews. Total maximum compensation for participation in the study will be $75. Each of the three telephone interviews should take approximately 90 minutes. You will be paid for the interviews even if you cannot or will not answer all of the questions you are asked.

You will not be charged for the three telephone interviews specific to the study. However, participation in the study will not free you from the need to pay any fees that you would ordinarily be charged for VA methadone/LAAM treatment.

VI. CONFIDENTIALITY

You are a patient in the VAMHC. The fact that you are participating in this research study and the results of medical text for this study will be included in your medical record. Your medical and research records will be kept strictly confidential to the fullest extent permitted by law.

Your study information will be stored on a password-protected computer and in a locked filing cabinet at the Menlo Park VA. These computers and locked file cabinets are located in the study office. They are only available to the study staff. Your social security number will be stored separately from your name and any of your research records. Any personal identification will be removed from your records as soon as all information has been collected. If the results of this study are published in the scientific literature, your identity will not be revealed.
A Certificate of Confidentiality has been received by the Menlo Park VA study center from the Department of Health and Human Services. This certificate protects investigators from being forced to release research data. This protection, however, is not absolute. It does not, for instance, apply to state requirements to report certain communicable diseases or child abuse.

VII. RIGHT TO WITHDRAW

Participation in this study is voluntary. You are not obligated to participate in this research. You are free to withdraw your consent at any time. Refusal to participate will not affect your current or future medical care in any way at the Baltimore VA Medical Center or the University of Maryland at Baltimore, University of Maryland Medical System of the VAMHCS. You will be told of any significant new findings that develop during the study, which may affect your willingness to participate in the study.

VIII. UNIVERSITY STATEMENT

The University is committed to providing subjects of its research all rights due them under State and Federal law. You give up none of your legal rights by signing this consent form or by participating in the research project. Please call the Institutional Review Board (IRB) if you have questions about your rights as a research subject.

The research described in this consent form has been classified as minimal risk by the University of Maryland Institutional Review Board (IRB), a group of scientists, physicians and other experts. The IRB membership includes people who are not affiliated with the University and people who do not conduct research projects. The Boards decision that the research is of minimal risk does not, however, mean that the research is risk free. Generally speaking, you are assuming the risks of research participation, as discussed in the consent form. But if you are harmed as a result of negligence of a researcher, you can make a claim for compensation. If you believe you have been harmed through participation in this research study as a result of researcher negligence, you can contact the IRB for more information about claims procedures at:

Institutional Review Board
University of Maryland at Baltimore
685 West Baltimore St.
Baltimore, Maryland 21201
(410) 706-5037

If you have questions about your rights as a study participant or are dissatisfied at any time with any aspects of this study, you may also contact (anonymously if you wish):

Administrative Panels Office
Stanford University
Stanford, California (USA) 04305-5401
Telephone collect 1 (650) 723-2480

**NOT VALID WITHOUT THE
IRB STAMP OF CERTIFICATION**

**APPROVED**

M1174

University of Maryland Institutional Review Board

**Valid from 10/05/01 to 1/16/02**

______________________________
Subject's signature

Date:______________________________
___ I have read and understand the information on this form
___ The information on this form has been explained to me.

______________________________
Signature of Parent/Legally Appointed Guardian
*(When Applicable)*

Date:______________________________

______________________________
Signature of Investigator or Authorized
Representative obtaining informed consent

Date:______________________________

______________________________
Witness to Consent procedures
*(Optional unless subject is illiterate, or unable to sign)*

Date:______________________________

______________________________
Investigator reviewed consent form and is aware of
Subject enrollment.

Date:______________________________

NOTE: This Consent Form, with the original signatures, must be retained on file by the Principal Investigator. A copy must be given to the volunteer. A copy must also be placed in the patient's medical record, if applicable.
